# Regulatory T lymphocytes/Th17 lymphocytes imbalance in autism spectrum disorders: evidence from a meta-analysis

**DOI:** 10.1186/s13229-021-00472-4

**Published:** 2021-10-12

**Authors:** Pierre Ellul, Michelle Rosenzwajg, Hugo Peyre, Gwladys Fourcade, Encarnita Mariotti-Ferrandiz, Vincent Trebossen, David Klatzmann, Richard Delorme

**Affiliations:** 1 AP-HP (Assistance Publique-Hôpitaux de Paris), Robert Debré Hospital, Child and Adolescent Psychiatry Department, Paris University, 48 Boulevard Sérurier, 75019 Paris, France; 2 INSERM, Immunology-Immunopathology-Immunotherapy (i3), Sorbonne Université, Paris, France; 3 AP-HP, Hôpital Pitié-Salpêtrière, Biotherapy (CIC-BTi), Paris, France; 4 Robert Debré Hospital, UMR 1141, NeuroDiderot Inserm – Paris University, Paris, France; 5 Human Genetics and Cognitive Functions, Institut Pasteur, Paris, France

**Keywords:** ASD, Immunology, Peripheral blood, Regulatory T lymphocyte, Th17 lymphocytes

## Abstract

**Background:**

Immune system dysfunction has been proposed to play a critical role in the pathophysiology of autism spectrum disorders (ASD). Conflicting reports of lymphocyte subpopulation abnormalities have been described in numerous studies of patients with ASD. To better define lymphocytes abnormalities in ASD, we performed a meta-analysis of the lymphocyte profiles from subjects with ASD.

**Methods:**

We used the *PRISMA* recommendations to query PubMed, Embase, PsychoINFO, BIOSIS, Science Direct, Cochrane CENTRAL, and *Clinicaltrials.gov* for terms related to clinical diagnosis of ASD and to lymphocytes’ populations. We selected studies exploring lymphocyte subpopulations in children with ASD. The search protocol has been registered in the international *Prospective Register of Systematic Reviews* (CRD42019121473).

**Results:**

We selected 13 studies gathering 388 ASD patients and 326 healthy controls. A significant decrease in the CD4+ lymphocyte was found in ASD patients compared to controls [− 1.51 (95% CI − 2.99; − 0.04) *p* = 0.04] (*I*^2^ = 96% [95% CI 94.6, 97.7], *p* < 0.01). No significant difference was found for the CD8+ T, B and natural killer lymphocytes. Considering the CD4+ subpopulation, there was a significant decrease in regulatory T lymphocytes (Tregs) in ASD patients (*n* = 114) compared to controls (*n* = 107) [− 3.09 (95% CI − 4.41; − 1.76) *p* = 0.0001]; (*I*^2^ = 90.9%, [95% CI 76.2, 96.5], *p* < 0.0001) associated with an increase oin the Th17 lymphocytes (ASD; *n* = 147 controls; *n* = 128) [2.23 (95% CI 0.79; 3.66) *p* = 0,002] (*I*^2^ = 95.1% [95% CI 90.4, 97.5], *p* < 0.0001).

**Limitations:**

Several factors inducing heterogeneity should be considered. First, differences in the staining method may be responsible for a part in the heterogeneity of results. Second, ASD population is also by itself heterogeneous, underlying the need of studying sub-groups that are more homogeneous.

**Conclusion:**

Our meta-analysis indicates defects in CD4+ lymphocytes, specifically decrease oin Tregs and increase in Th17 in ASD patients and supports the development of targeted immunotherapies in the field of ASD.

**Supplementary Information:**

The online version contains supplementary material available at 10.1186/s13229-021-00472-4.

## Background

Autism spectrum disorders (ASD) define a heterogeneous group of neurodevelopmental disorders characterized by a deficit in social communication associated with restrictive, repetitive and stereotyped behaviors [1]. ASD affects about 1 in 54 people in the general population with a burden of 58 Disability Adjusted Life Years per 100,000 individuals [2]. The neurobiology of ASD remains largely unknown although many genetic associations, as well as complex gene-environment interactions have been reported [3, 4]. The immune system seems to play a crucial role in the etiology of ASD. Several studies linked ASD with personal/familial history of autoimmune disorders such as diabetes mellitus, celiac disease, autoimmune thyroiditis, rheumatoid arthritis, psoriasis, systemic lupus erythematosus [5, 6]. Post-mortem analysis of brain tissues from individuals with ASD has shown two main disrupted biological pathways: a down-regulation of the genes associated with synaptic functions and an up-regulation of immune-related genes such as the genes involved in the M2-microgial cell states, or in the interferon and cell signaling pathways [7, 8]. Alterations of the peripheral immune system have also been reported with quantitative and qualitative immune dysfunctions, specifically abnormal lymphocyte subpopulations [5].

B-, T- and NK-cells, are closely linked in a dynamic balance [9]. Any modification of one of these subpopulations affects the whole pattern of immune cells and therefore, affects the homeostasis of the human body [10]. Lymphocytes, well known for their roles against pathogens, have also several functions devoted to organ-specific homeostasis [9]. For example, regulatory T cells (Tregs), a subset of T lymphocytes, are involved in the maintenance of the immune system homeostasis [10], tissue regeneration [11] and in maternal and fetal cardiomyocyte proliferation during pregnancy [12]. The immune system has also a critical role in neurodevelopment and associated functions [13]. For example, cytokines have a central physiological role in brain development, cognitive functions and behavioral regulation such as interleukin-1 beta in synapses formation and contextual learning, interleukin 4 in promoting neurogenesis and spatial learning or interleukin 17a (IL-17a) for synaptic plasticity and the regulation of anxiety (for a complete review see [14, 15]). It should also be cited the fundamental role of the complement in synaptic pruning during normal brain development [16]. Thus, an immune system disruption may affect the structural micro-architecture of the brain and the underlined cognitive functions [17].

In ASD, several studies reported changes in peripheral blood lymphocyte subsets such as reduced total number of lymphocytes, impairment of the CD4/CD8 T cell ratio, a defective activation of T cells, an increased number of the natural killer (NK) cells, an imbalance of the Th1/Th2 cytokines but also some aberrations in cytotoxicity related to NK cells (for review see [18]). Additional reports showed also the presence of autoantibodies directed toward central nervous system (CNS) proteins, suggesting a deregulation of B lymphocytes [19]. In animal models of ASD, similar immune alterations were reported, showing an association between abnormal CNS development and ASD-like behaviors in pups [5]. For example, higher proportion of pro-inflammatory lymphocytes or altered NK cell activity induced abnormal cortical development, stereotyped behaviors, and social communication deficit in mice [20].

### Aims of the study

These preliminary findings support a striking link between ASD and immune dysfunctions. However, studies on lymphocyte subpopulation in ASD have reported conflicting results. We thus, conducted a systematic review and meta-analysis to identify specific abnormal lymphocyte imbalance in ASD.

## Methods and materials

### Search strategy

The protocol for the present systematic review/meta-analysis was registered on the international Prospective Register of Systematic Reviews PROSPERO (protocol number: CRD42019121473). The systematic review and meta-analysis were conducted and reported following the Preferred Reporting Items for Systematic review and Meta-Analysis (PRISMA) recommendations [21]. The following electronic databases were searched with no restriction in terms of language, type of document, or date: PubMed (MEDLINE), Embase, PsychoINFO, BIOSIS, Science Direct, and Cochrane CENTRAL. The following search terms/syntax were used for Pubmed: (autism[tiab] OR Asperger[tiab] OR autism spectrum disorder[tiab] OR pervasive developmental disorder[tiab] OR autistic[tiab] OR ASD[tiab] OR PDD[tiab]) AND (lymphocytes[tiab] OR CD3[tiab] OR T cells[tiab] OR Natural Killer[tiab] OR CD56 lymphocyte[tiab] OR CD4 lymphocyte[tiab] OR CD8 lymphocyte[tiab] OR B lymphocyte[tiab] or B cells[tiab] OR CD20[tiab] OR Regulatory T lymphocyte[tiab] OR Foxp3[tiab] OR Th1 lymphocyte[tiab] OR Th2 lymphocyte[tiab] OR T9 lymphocyte[tiab] OR Th17 lymphocyte[tiab] OR GATA 3[tiab] OR T-bet[tiab] OR RORgt[tiab] OR γδ T cells[tiab] OR unconventional T cells[tiab] OR MAIT[tiab]).

The search terms/syntax were adapted accordingly for the other databases. Reference lists of the retained articles and relevant review articles were hand-searched to retrieve any additional pertinent reports not detected via the electronic database search. Furthermore, we used the Clinicaltrials.gov website to identify any relevant studies not yet published as full text articles at the time of the search. The last search was completed on January 2021.

### Selection of the relevant articles

We only included in our systematic review case–control studies which included participants with a mean age under 18 years old with a diagnosis of Autism, Asperger, pervasive developmental disorder (PDD) or ASD, in which the authors explored one or more lymphocyte subpopulations.

### Selection of studies and data extraction

The eligibility process was conducted in two separate stages: (1) two researchers (PE and HP) independently screened all non-duplicated references initially retrieved as potentially pertinent and excluded those clearly not pertinent based on title or abstract. A final list was agreed with discrepancies resolved by consensus between the two authors. When consensus was not reached, a third, senior researcher (RD) acted as arbitrator; (2) the full-text version of the articles passing stage 1 screening were downloaded and assessed for eligibility by the two researchers, independently. Discrepancies were resolved by consensus between the two researchers and, if needed, the third senior researcher acted as arbitrator. When required, corresponding authors were contacted to clarify study eligibility and gather unpublished data.

### Statistical analyses

The difference between each study was first calculated as the lymphocyte value in the ASD group minus the lymphocyte value in the control group, divided by the pooled pre-test standard deviation with a bias adjustment [22]. The difference for each study was then combined using the inverse variance method. Given the inherent heterogeneity of studies, random-effects models were used. Heterogeneity was statistically assessed by estimating *I*^2^, *τ*^2^ and *p*-value of heterogeneity test. The statistic *I*^2^ was calculated to estimate between-study heterogeneity. I^2^ represents the percentage of variance due to between-studies factors rather than sampling error [23]. *τ*^2^ statistic provides a measure of the variability of the effect estimate across studies in a random‐effects model [24]. Analyses were performed in R by use of the *metabin* command from the package *meta*.

## Results

### Characteristics of studies included in the meta-analysis

**Fig. 1:**
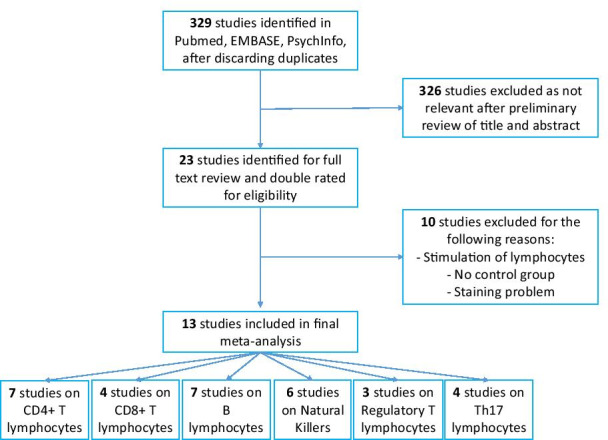
Flowchart of studies included

Based on our selection criteria (Fig. 1 & Additional file 1: table 1), we included 13 studies gathering 388 individuals with autism and 326 healthy controls [25–37]. Seven studies analyzed the CD4+ T lymphocytes (LT4) (194 cases and 152 controls), four the CD8+ T lymphocytes (LT8) (124 cases and 83 controls), three the Tregs subpopulation (114 cases and 107 controls), seven the B lymphocytes (LB) (194 cases and 152 controls), six the NK cells (176 cases and 138 controls) and four the Th17 T-cells (Th17) (147 cases and 128 controls). No data were available for the others lymphocyte subpopulations including in our search strategy. Details of included studies can be found in Additional file 2: table 2, Additional file 3: table 3, Additional file 4: table 4, Additional file 5: table 5, Additional file 6: table 6, Additional file 7: table 7.

### Results of the meta-analysis

**Fig. 2:**
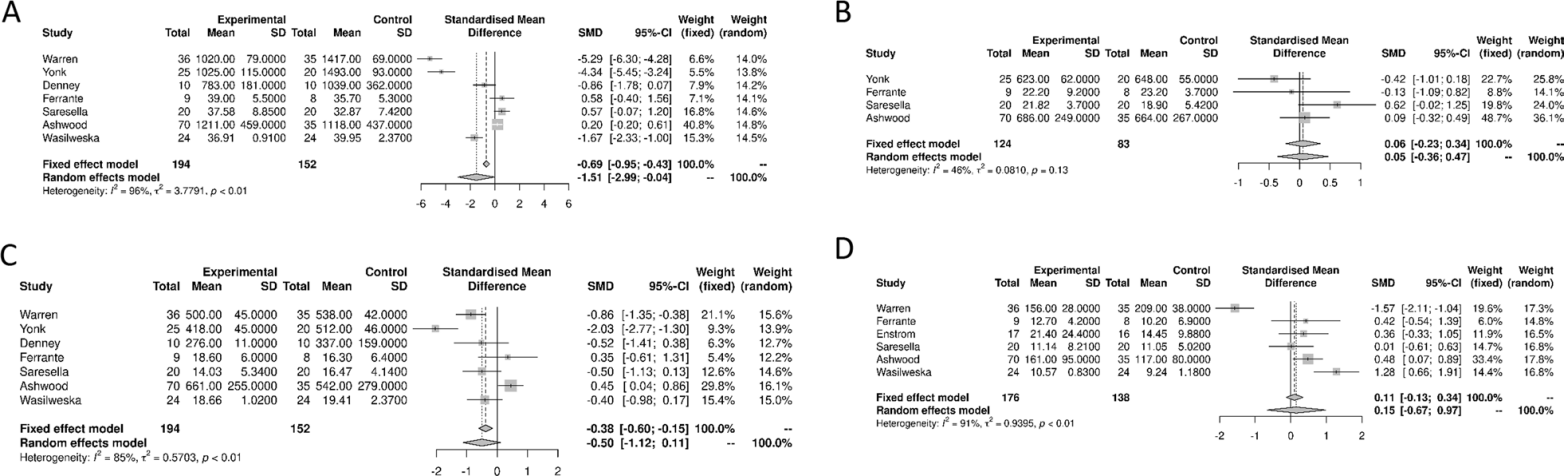
Forest plots of the distinct lymphocyte subpopulations in autism spectrum disorders compared to controls. **A** T CD4+ lymphocytes; **B** T CD8+ lymphocytes; **C** B Lymphocytes; **D** natural killer cells

We first explored the association between the CD4+ T lymphocytes, CD8+ T lymphocytes, LB and NK cells with ASD (Fig. 2). We observed a significant decrease in the peripheral blood CD4+ T lymphocytes in ASD compared to healthy controls [− 1.51 (95% CI − 2.99; − 0.04) *p* = 0.04] with a significant heterogeneity (*I*^2^ = 96% [95% CI 94.6, 97.7], *p* < 0.01). We observed no significant differences between ASD and controls for the peripheral blood CD8+ T lymphocytes, LB and NK cells [0.05 (95% CI − 0.36; 0.47) *p* = 0.8; − 0.50 (95% CI − 1.12; 0.11) *p* = 0.11; and 0.15 (95% CI − 0.67; 0.97) *p* = 0.7, respectively].

**Fig. 3:**
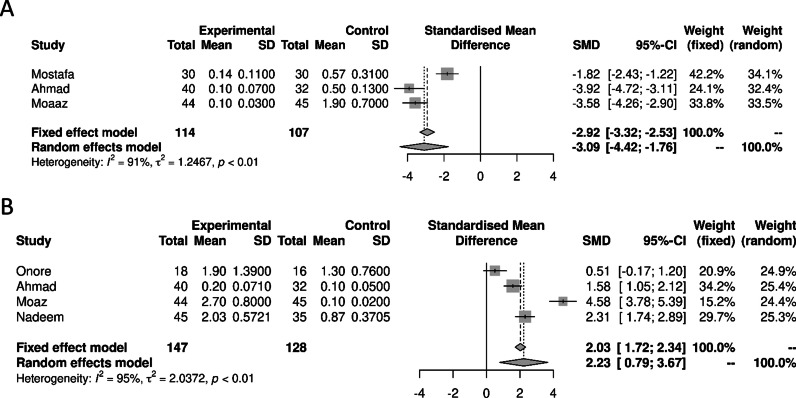
Forest plots of the distinct lymphocyte subpopulations in autism spectrum disorders compared to controls. **A** Regulatory T lymphocytes; **B** Th17 Lymphocytes

Considering Tregs and Th17 subpopulations (Fig. 3), we observed a significant decrease in Tregs cells [− 3.09 (95% CI − 4.41; − 1.76) *p* = 0.0001] in ASD peripheral blood cells compared to controls (*I*^2^ = 90.9%, [95% CI 76.2, 96.5], *p* < 0.0001). We also found a significant increase oin the Th17 cells in ASD [2.23 (95% CI 0.79; 3.66) *p* = 0.002] [*I*^2^ = 95.1% [95% CI 90.4, 97.5], *p* < 0.0001] compared to controls. Due to methodological limits, we were unable to calculate the Treg/Th17 ratio. Due to lack of data we were also unable to study the other lymphocyte subpopulations included in the search protocol.

## Discussion

Our meta-analysis pointed toward abnormalities of the number of peripheral CD4+ lymphocyte and especially Th17 and Tregs cells in patients with ASD.

Th17 are pro-inflammatory CD4+ T cells that are associated with inflammatory immune response in infection or autoimmune disorders [46], but may also regulate brain architecture and behaviors [38]. Tregs are a subset of CD4+ T cells critical for the maintenance of the immune system homeostasis and peripheral tolerance. Brain Tregs cells also play an important role in myelination through regeneration/protection mechanisms [13]. Thus, the biological properties of Th17 cells and Tregs in the brain might be related to the observed pathophysiology of ASD.

The differentiation of naïve T cell precursors into Tregs or Th17 depends on the cytokine environment. Indeed, TGF-B and IL-2 induce Tregs whereas TGF-B and IL6, Th17 cells. Those cytokines act in part through the up-regulation (for Th17) or down-regulation (for Tregs) of the *mammalian target of rapamycin* (mTOR) complex [39, 40]. In neurons or Purkinje cell, mTOR complex has a major role in brain development and neuron homeostasis [41] and is severely affected in ASD related syndromes such as the tuberous sclerosis. In peripheral blood mononuclear cells, overexpression of the mTOR pathway in patients with ASD [42] has recently been associated with increased expression of the Th17 specific transcription factor (Rorγt) and reduced expression of the Treg transcription factor (Foxp3). Thus, the transcription factor anomalies found in ASD are also consistent with our results showing a decrease in Tregs and increase in Th17.

From a clinical point of view, our results are also relevant for the co-morbidities observed in ASD. First, there is a significant increase in the prevalence of atopic diseases (including allergy and asthma) among patients with ASD [43]. If Th2 lymphocytes orchestrate immune responses in atopic diseases [44], Tregs is of major importance in maintaining tolerance to several antigens, thus playing a central role in allergy [45]. For example, a relative or absolute defect in Tregs in airways bias immune response toward Th2 leading to allergic inflammatory diseases [46]. In line with these data, unstable Tregs phenotype has been found in asthmatic patients and is associated with a more severe disease. Collectively, these data pointed to a central role of Tregs in atopic diseases [46]. Thus, we postulate that the decrease in Tregs observed in ASD patients could be, at least in part, responsible for this observed comorbidity. Secondly, individuals with ASD have long been associated with an increase in gastrointestinal symptoms (diarrhea/constipation) and intestinal permeability, related to an alteration in the microbiota composition [47]. Indeed, recent meta-analysis found low Bifidobacterium and higher proportion of Bacteroides, Parabacteroides, Clostridium phyla in ASD patients [48]. Microbiota composition is closely intricated with lymphocytes subtype development and function (especially Tregs and Th17). For example, while segmented filamentous bacteria promote the development of Th17 cells, Bifidobaberitum induced Tregs [49]. Although no formal causal role can be established, we hypothesized that the decrease in Tregs and increase in Th17 found may be related to microbial dysbiosis.

Interestingly, a similar lymphocyte pattern has been found in several different mouse models of ASD, suggesting that imbalance of Treg and Th17 lymphocytes may be an important common physiopathological pathway in autism (see Additional file 8: table 8). Unfortunately, due to the low number of studies and the various gating strategies, we were unable to carry out analyses of these animal studies. Interestingly in a mouse model of ASD, IL-17 injected directly in the brain transiently reversed the ASD-like symptomatology, suggesting a possible physiological role of IL-17a lymphocytes in the CNS, later confirmed by a recent study [50]. These data may seem contradictory to our results but may also mean that the lymphocyte profile observed in the periphery is different from that in the brain. As seen in MIA model, IL-17a secreted by mother during pregnancy induce ASD. Thus, another possible explanation is that IL-17a may have different effects on brain development depending on the timing of exposure. Our findings highlight the need for further research to better understand the intriguing role of Treg, Th17 and IL-17 in ASD.

### Limitations

Our results should be taken in light of the strengths and limits of the study. Several factors inducing heterogeneity should be considered. The different lymphocytes populations are separated by flow cytometry and the staining strategy is not precisely the same in all the studies, indicated that we may not be looking exactly at the same cells. This difference in the staining method might be responsible for a part in the heterogeneity of results. It is also important to note that in the general population, the frequency of the different subgroups within T cells can vary from a normal range of 22 to 93% of the total lymphocytes [51]. Despite this, we still have found differences and comparing such variable populations reinforces the power of our results [52, 53]. Lastly, ASD population is also by itself heterogeneous, underlying the need of studying sub-groups that are more homogeneous. Because we found significant differences despite factors inducing heterogeneity, our main results could be considered as robust and relevant. Results on lymphocyte subpopulation—Tregs, Th17—should also be taken with precaution. Indeed, because those populations are more precise, we used more stringent staining protocols. As a consequence, we limit the number of studies per analysis, losing power but increasing sensibility. Because no study screened them, it is also important to note that several lymphocytes subpopulation (see research algorithm) were not included in this meta-analysis and may have a role to play in the pathophysiology of ASD. Furthermore, due to lack of data, we were not able to analyze data from other tissues than peripheral blood. Therefore, it is important to note that our work focuses on peripheral populations and does not prejudge immune changes within the CNS. For the strengths of our study, by using the criteria of the PRISMA statement, we performed a systematic search in several databases, without language restrictions, as well as in Clinicaltrials.gov. We have also contacted authors to gathered additional unpublished data, limiting the publication bias. Thus, we have included an important number of studies, with more than 700 subjects.

## Conclusion

More than immunological abnormalities in ASD, we claim for more homogeneous and precise immunological studies in the field of psychiatry. In line with this idea, further large-scale studies on ASD patients should be done. Next, experimental animal studies should be performed to delineate the type of immune intervention aimed in ASD. We can hope that these findings will pave new avenues for therapeutic strategies involving the immune-modulation in ASD.

## Supplementary Information

**Additional file 1.** Studies rejected with reasons.

**Additional file 2.** Characteristics of CD4+ T lymphocytes studies.

**Additional file 3.** Characteristics of CD4+ lymphocytes studies.

**Additional file 4.** Characteristics of B lymphocytes studies.

**Additional file 5.** Characteristics of natural killers studies.

**Additional file 6.** Characteristics of Tregs studies.

**Additional file 7.** Characteristics of Th17 studies.

**Additional file 8.** Characteristics of mice studies with lymphocytes subpopulations.

## Data Availability

All data generated or analyzed during this study are included in this published article and its supplementary information files.

## Abbreviations

ASD: Autism spectrum disorders
CNS: Central nervous system
LB: B lymphocytes
LT4: CD4+ T lymphocytes
LT8: CD8+ T lymphocytes
mTOR: *Mammalian target of rapamycin*
PDD: Pervasive developmental disorder
Th17: Th17 T-cells
Treg: Regulatory T lymphocytes
