# Supplementary material for: Regulatory T lymphocytes/Th17 lymphocytes imbalance in autism spectrum disorders: evidence from a meta-analysis
Source: Mol Autism. 2021 Oct 12;12:68. doi: 10.1186/s13229-021-00472-4 (PMC8507168; doi:10.1186/s13229-021-00472-4)
Supplement: Supplementary file 1 — Additional file 1. Studies rejected with reasons. [file 13229_2021_472_MOESM1_ESM.docx]

| **Studies** | **Reason of reject** |
| --- | --- |
| Warren, 1986 | Stimulated lymphocytes |
| Warren, 1990 | Stimulated lymphocytes |
| Plioplys, 1994 | No control group |
| Warren, 1995 | Staining not precise enough to properly determine the subpopulation studied |
| Gupta, 1998 | Staining not precise enough to properly determine the subpopulation studied |
| Sweeten, 2003 | Staining strategy not precise enough |
| Ashwood, 2006 | Staining strategy not precise enough in blood |
| Ahmad, 2018 | Staining strategy not precise enough in blood |
| Bennabi, 2019 | Data on adults |
| Basheer, 2019 | Data not available even after contacting the authors |

Supplementary table 1: Studies rejected with reasons
