# Supplementary material for: Regulatory T lymphocytes/Th17 lymphocytes imbalance in autism spectrum disorders: evidence from a meta-analysis
Source: Mol Autism. 2021 Oct 12;12:68. doi: 10.1186/s13229-021-00472-4 (PMC8507168; doi:10.1186/s13229-021-00472-4)
Supplement: Supplementary file 5 — Additional file 5. Characteristics of natural killers studies. [file 13229_2021_472_MOESM5_ESM.docx]

Supplementary table 5: Characteristics of natural killers studies
