# Supplementary figures and images for: Regulatory T lymphocytes/Th17 lymphocytes imbalance in autism spectrum disorders: evidence from a meta-analysis
Source: Mol Autism. 2021 Oct 12;12:68. doi: 10.1186/s13229-021-00472-4 (PMC8507168; doi:10.1186/s13229-021-00472-4)

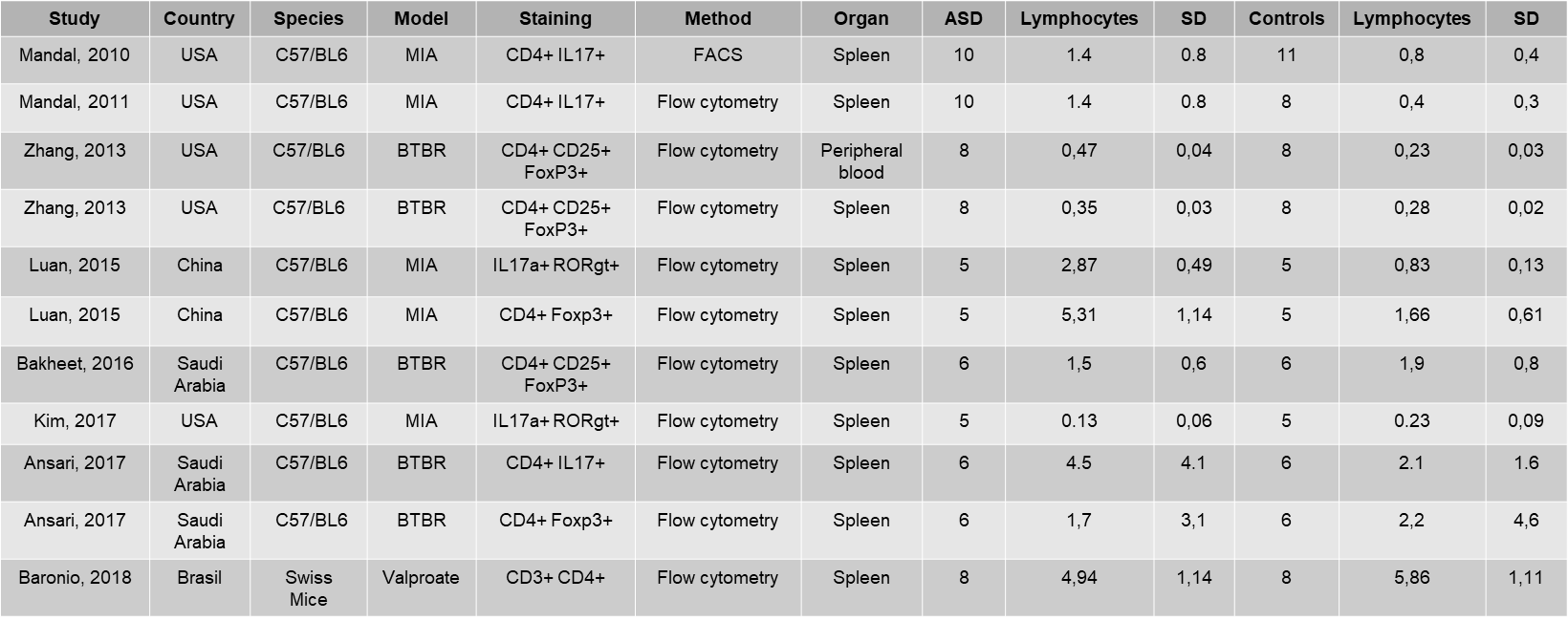
 Supplementary table 8: Characteristics of mice studies and lymphocytes subpopulations

Supplement: Supplementary file 8 — Additional file 8. Characteristics of mice studies with lymphocytes subpopulations. [file 13229_2021_472_MOESM8_ESM.docx]
